# A Simplified Method for the Isolation of Extracellular Vesicles from Probiotic Bacteria and Their Characterization

**DOI:** 10.3390/ijms26031058

**Published:** 2025-01-26

**Authors:** Harshal Sawant, Ji Bihl, Alip Borthakur

**Affiliations:** 1 Department of Biomedical Sciences, Joan C. Edwards School of Medicine, Marshall University, Huntington, WV 25701, USA; sawantha@marshall.edu (H.S.); bihlj@marshall.edu (J.B.); 2 Department of Clinical and Translational Sciences, Joan C. Edwards School of Medicine, Marshall University, Huntington, WV 25701, USA

**Keywords:** probiotic extracellular vesicles, *Escherichia coli* Nissle, *Lactobacillus acidophilus*, ultracentrifugation, precipitation, nanoparticle-tracking analysis

## Abstract

Probiotic bacteria are normal inhabitants of a healthy human gut, conferring multiple beneficial effects on the gut and beyond. Under various disease states, the abundance and diversity of beneficial bacteria are significantly decreased, a process called dysbiosis. Among the intra- and extracellular components of probiotics, the extracellular vesicles (EVs) secreted by them have recently garnered significant attention as potential mediators of probiotics’ effects on host health. Further, these nanosized particles that encapsulate a wide range of bioactive molecules (proteins, lipids, RNA, and DNA) are standing out as key factors that could mediate gut microbiota–host communication and confer ameliorating effects in experimental inflammatory, metabolic, and cardiovascular disease models. However, a standard protocol of EV isolation from probiotic bacteria, not varying from lab to lab, must be established to achieve consistency in the experimental results in these pre-clinical models. Our current study compared two commonly used methods for EV isolation from biological samples, ultracentrifugation and precipitation, to develop a standard protocol for isolating EVs from the probiotics *Lactobacillus acidophilus* (LA)*,* a Gram-positive bacterium, and *Escherichia coli* Nissle (EcN)*,* a Gram-negative bacterium. The ultracentrifugation method gave ~1.5-fold higher EV yield for both LA and EcN compared to the precipitation method. Further, EcN released a higher level of EVs compared to LA. EVs were quantified and characterized by nanoparticle-tracking analysis (NTA) and by measuring the specific surface biomarkers using Western blot. Here, we describe our standardized step-by-step protocol for isolating EVs from probiotic bacteria and their characterization.

## 1. Introduction

Like mammalian cells, bacteria release nano-sized (100–1000 nm) membrane vesicles into the extracellular environment either constitutively or in a regulated manner. These bacterial extracellular vesicles (EVs) are spherical structures with a proteolipid bilayer and are enriched with bioactive proteins, lipids, nucleic acids, and virulence factors [1]. The EVs are produced as an end product of secretion by pathogenic, non-pathogenic, and probiotic bacterial species that confer multiple health benefits on human health. EVs were first reported in *Escherichia coli*, but their existence in Gram-positive bacteria gained attention only recently [2]. Bacterial EVs are established mediators of intracellular signaling, stress tolerance, horizontal gene transfer, immune stimulation, and pathogenicity [3]. Due to their outer membrane structure and composition differences, Gram-negative and Gram-positive bacteria produce EVs via different mechanisms. For example, Gram-negative bacteria have a thin proteoglycan layer and an additional outer membrane, leading to distinct EV components like lipopolysaccharide (LPS). Gram-positive bacteria with multiple proteoglycan layers and lacking the outer membrane produce EVs with different protein and lipid profiles [3].

Until recently, bacterial EVs were described as carriers of virulent factors to the host cells, thereby mediating bacterial pathogenesis. Consequently, the significant role of EVs in the virulence of various life-threatening pathogens has been extensively documented. Conversely, the beneficial health effects associated with EVs released by non-pathogenic microorganisms, such as probiotics, are less known [4]. The probiotics are live microorganisms that, when administered in adequate amounts, confer a health benefit on the host [5], typically by improving or restoring the gut flora [6]. Probiotics are found in fermented foods, dietary supplements, and certain dairy products, but importantly, most of them are normal residents of the human gut. The most common types of probiotic bacteria include species from the *Lactobacillus* and *Bifidobacterium* genera. However, the list of next-generation probiotics is growing, with important members like *Faecalibacterium prausnitzii* and *Akkermansia muciniphila* showing promising health benefits [7,8].

For the past few years, interest in probiotics-derived EVs as a very promising therapeutic strategy for intestinal and extraintestinal diseases has been rapidly growing. The anti-inflammatory effects and protection of the intestinal epithelial barrier function by the EVs released by *E. coli* Nissle 1917 have been demonstrated in both in vitro and in vivo experiments [9,10]. Additionally, it has been shown in recent studies that bacteriocins, capable of killing other pathogenic bacteria, could be delivered by EVs released by *L*. *acidophilus* ATCC 53544 [11]. Beyond the intestine, probiotics-derived EVs have been shown to exert ameliorating effects in cardiovascular complications [12], cancers [13], and various neurological disorders [14,15]. For example, *Lactobacillus paracasei* EVs have been shown to cross the blood–brain barrier and affect neuronal cells, thereby modulating amyloid-induced changes in the mouse brain [16]. Similarly, by inhibiting the extracellular signal-regulated kinase (ERK) and p38 signaling pathways, the EVs from kimchi-derived *Lactobacilli* could block the inflammatory response in LPS-stimulated microglia [17]. Additionally, probiotic EVs are being explored as vehicles for targeted delivery approaches [18,19]. However, the clinical utility of probiotic EVs is hindered by several challenges in pre-clinical experimental models. Major factors that lead to inconsistency in experimental results could be the lack of standard protocols for culturing specific bacterial species and isolating and characterizing their EVs [20]. Unstandardized techniques for isolating EVs create challenges in reproducibility, purity, and consistency, complicating the comparison of results across studies. Variability in methods, such as ultracentrifugation or size-exclusion chromatography, often leads to contamination with non-vesicular components or the selective isolation of certain EV types [21]. A uniform and simplified method with details is needed to troubleshoot issues regarding quality and quantity variations in EV isolation. This will support the customization of EV isolation depending on the probiotic type and source and unlock the therapeutic and research potential of probiotic EVs [21,22].

In this brief report, we describe a step-by-step protocol for isolating and characterizing the EVs produced by *Lactobacillus acidophilus* (LA), a Gram-positive probiotic, and *E. coli* Nissle 1917 (EcN), a Gram-negative probiotic. We also briefly describe the methods for culturing LA and EcN that have been standardized for optimal EV yield.

## 2. Results

### 2.1. Ultracentrifugation Method Leads to Higher EV Yield

**Figure 1:**
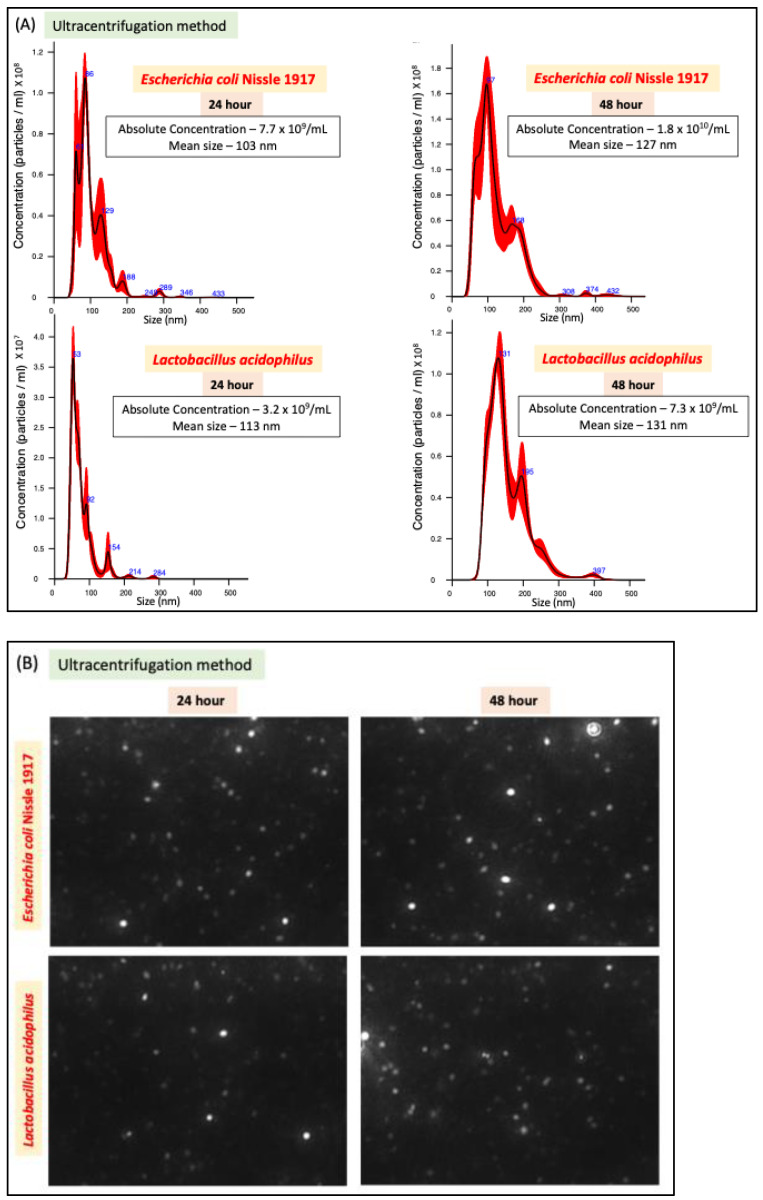

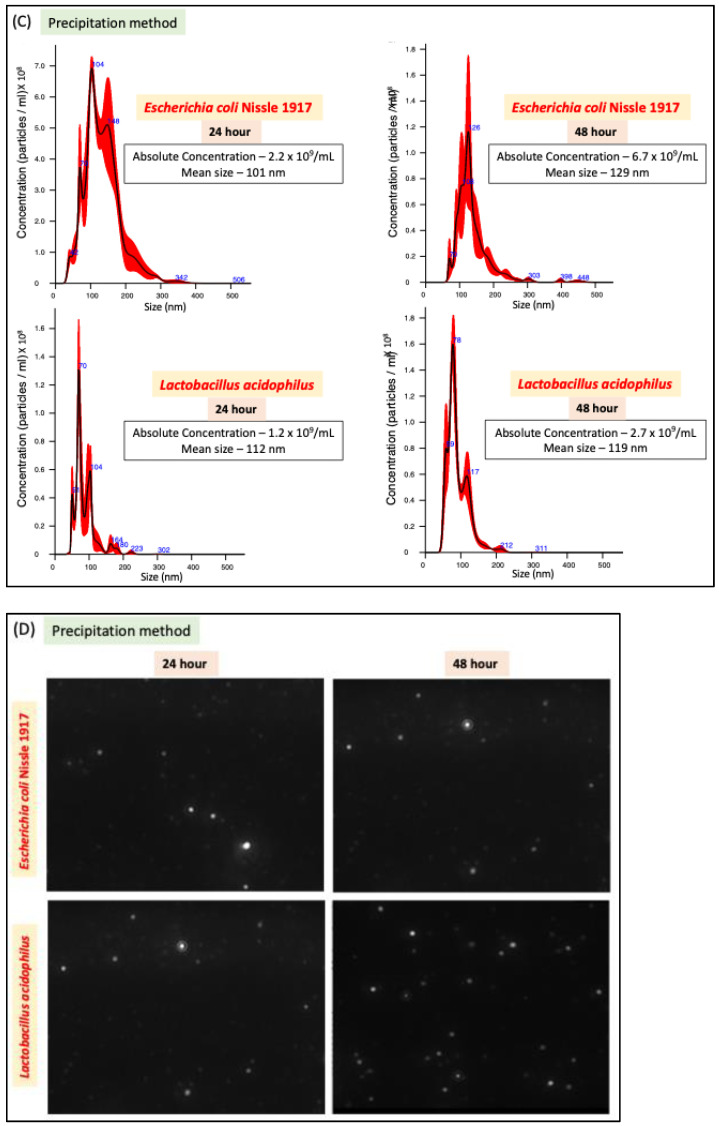

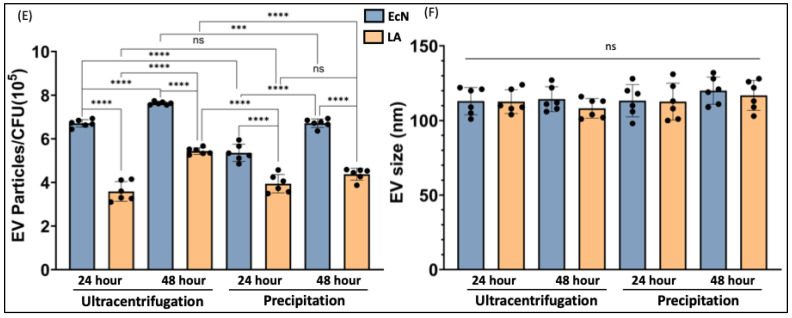
NTA of EcN-EVs and LA-EVs for two time points (24 and 48 h) and two methods of EV isolation (ultracentrifugation and precipitation method). (**A**) NTA data in graph format for size vs. concentration along with (**B**) video images of EcN-EVs in motion via light scatter mode of NTA. Magnification-20X. (**C**) NTA data in graph format for size vs. concentration along with (**D**) video images of LA-EVs in motion via light scatter mode of NTA. Magnification-20X. (**E**) Data analysis of EV particles/CFU for EcN and LA. (**F**) Data analysis of EV particle size for EcN and LA. Data represent mean ± SEM. *** *p* < 0.001, **** *p* < 0.0001, and ns = not significant between groups as indicated.

We compared two methods of bacterial EV preparation: ultracentrifugation and precipitation. The ultracentrifugation method gave ~1.5-fold higher EV yield for both LA and EcN compared to the precipitation method (Figure 1A–D). We also noted that EcN releases a higher amount of EVs compared to LA (Figure 1E) with no size differences (Figure 1F). For both bacteria, EV release marginally (1.5 fold) but significantly increased when the culture time was increased from 24 h to 48 h. (Figure 1E).

### 2.2. Markers of EVs from LA and EcN

**Figure 2:**
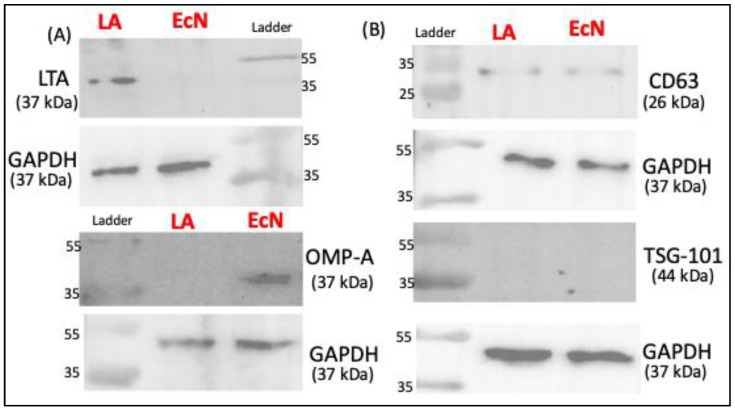
Western blot data showing protein bands for protein of interest. (**A**) Bacterial-specific EV markers. (**B**) Well-known extracellular vesicles markers.

Since the ultracentrifugation method showed higher EV yield, we next used Western blot to characterize the markers of LA-EVs and EcN-EVs isolated by this method. EcN-EVs were positive for outer membrane protein A (OMP-A) antibody whereas LA-EVs were stained by Lipoteichoic acid (LTA) antibody, suggesting that OMP-A and LTA, respectively, could be specific markers for Gram-negative and Gram-positive bacterial EVs. The presence of common eukaryotic EV markers (TSG-101 and CD63) was also assessed. CD63 showed faint bands while TSG-101 was not detected in both probiotic EVs (Figure 2B).

## 3. Discussion

In this study, we attempted to standardize a protocol for isolating extracellular vesicles (EVs) from two probiotic bacterial species with demonstrated health benefits on hosts. Although the most commonly used probiotic bacteria are species from the Gram-positive *Lactobacillus* and *Bifidobacterium* genera, several Gram-negative bacterial species are known to exhibit probiotic properties. In this regard, Gram-negative and Gram-positive bacteria differ in their outer membrane structure and composition, and therefore, these two groups are known to produce EVs via different mechanisms. Thus, in the current study, it was quite logical to choose *Lactobacillus acidophilus* (LA), a Gram-positive probiotic, and *E. coli* Nissle 1917 (EcN), a Gram-negative probiotic, to determine whether the same protocol works for both in terms of yield and quality, despite their differential mechanisms of EV production. Both LA and EcN are known to have multiple health benefits as reported from several clinical and experimental studies [23,24,25].

Initially, we sought to compare two methods, ultracentrifugation and precipitation, commonly used to isolate EVs from eukaryotic cells and biological fluids. The relative efficacy of these two methods in isolating exosomes (a type of EVs) from human biological samples has been reported [26,27]. However, there have been no comparative studies to determine the relative efficiency of these two methods for isolating bacterial EVs. Although the precipitation method appears to be relatively simpler for isolating EVs from large volumes of culture supernatant, the purity of these preparations could be greatly affected due to the co-precipitation of other molecules [28,29]. Mechanistically, the EVs isolated from the probiotic *Lactobacillus plantarum* (strain WCFS1) by precipitation method enhanced the immune protection of enterococci infection in *C. elegans* [29]. On the other hand, in a different experimental model, the EVs isolated from the same strain (WCFS1) of *L. plantarum* by a combination of ultrafiltration and ultracentrifugation were less efficient than the EVs from *L. rhamnosus* GG (LGG) in clearing pathogen infection in a mouse model of sepsis [30]. Importantly, the *L. plantarum* (WCFS1) EVs reported in these studies from two different research groups differed in yield and particle size. This lack of uniformity and reproducibility of EV isolation protocols from lab to lab poses a great hindrance in translating the results of preclinical models to studies in clinical settings. Therefore, our attempt to standardize a simplified method of isolating bacterial EVs as reported here will be of great addition toward the utilization of bacterial EVs in clinical studies.

Our results showed that the EV yield from both probiotics was higher with the ultracentrifugation method compared to the precipitation method with an additional advantage of being less time-consuming. Also, the particle size range of isolated EVs for both LA and EcN was more uniform (less scattered) with the ultracentrifugation method compared to the precipitation method. Our findings that the ultracentrifugation method has greater efficiency than the precipitation method in isolating bacterial-EVs are consistent with a previous report [31]. A previous study showing better efficacy of the precipitation method over ultracentrifugation in isolating EVs from bodily fluid (plasma) [26] suggests that the efficiency of EV isolation could depend on the biological material from which they are being isolated. Doubling the bacterial culture time from 24 h to 48 h did not show a proportionate increase in EV yield for both probiotics. This is because beyond the log phage, bacterial growth is not exponential and reaches the saturation stage. Therefore, scaling up the culture volume, instead of prolonging the culture time, appears to be the better choice to have a higher yield of bacterial EVs. Whether growing bacteria for a longer time alters the cargo contents of their EVs merits detailed investigation.

EcN and LA-produced EVs were of similar particle size, although the EV yield was higher for EcN than LA. Whether this differential yield is due to differences in the EV release pattern from Gram-positive and Gram-negative bacteria needs to be ascertained in studies utilizing multiple strains of both types. OMP-A and LTA have previously been shown as specific markers, respectively, for other species of Gram-negative and Gram-positive bacteria [32]. We also used some common EV markers and found that EcN and LA-EVs do not express TSG-101 on their surface [33] and weakly express CD63.

The limitation of our study is that we used only one each of Gram-positive and Gram-negative probiotics to compare their EVs with respect to yield, particle size, and surface markers. However, this does not critically affect our primary aim of this study to standardize and describe a protocol for the isolation of EVs from probiotic bacteria. Establishing ultracentrifugation as more efficient than the precipitation method for bacterial EV isolation is another important aspect of this study.

## 4. Materials and Methods

### 4.1. Materials Required

(1) *Escherichia coli Nissle 1917* was obtained from Ardeypharm (GmbH, Herdecke, Germany).
(2) *Lactobacillus acidophilus* (strain 4356) was procured from the American Type Culture Collection (ATCC, Manassas, VA, USA).
(3) De Man, Rogosa and Sharpe (MRS) broth and MRS Agar: cat #69966, Millipore Sigma, St. Louis, MO, USA.
(4) Luria–Bertani (LB) broth and LB Agar: cat #L3397, Millipore Sigma, St. Louis, MO, USA.
(5) Incubators and shakers.
(6) Sorvall LEGEND XTR centrifuge: Thermo Scientific, Waltham, MA, USA.
(7) Filtration system (0.22 µM): cat# V25022, CellPro premium labware, Fort Lauderdale, FL, USA.
(8) Amicron Ultra-15 Centrifugal filter units: cat# UFC905024, Millipore Sigma, St. Louis, MO, USA.
(9) Ultracentrifuge Sorvall MX 120+: Thermo Scientific, Waltham, MA, USA.
(10) Total exosome isolation (from cell culture media): cat# 4478359, Invitrogen, Waltham, MA, USA.

### 4.2. Protocol

(1) Bacterial culture: *Lactobacillus acidophilus* (LA) was grown overnight at 37 °C in four culture tubes, each containing 50 mL of MRS broth. Two colonies from an MRS Agar plate were inoculated into each tube and grown without shaking. *Escherichia coli* Nissle 1917 (EcN) was grown overnight at 37 °C in two 500 mL flasks, each containing 100 mL of LB broth. Two colonies from an LB Agar plate were inoculated into each flask and grown with shaking at 150 rpm.
(2) After the bacteria were grown overnight, LA suspensions in 4 tubes and EcN suspensions in 2 flasks were combined together. The optical densities at 600 nm (OD_600 nm_) of the combined suspensions were measured to calculate the colony-forming unit (CFU) of each bacterial culture to be used for EV isolation.
(3) The bacterial suspensions were then centrifuged at 4000 rpm (3745× *g*) for 20 min at 4 °C to pellet down the bacteria.
(4) Supernatants were collected and filtered through 0.22 µm filtration systems to obtain bacteria-free culture supernatants.
(5) Large volumes (200 mL) of culture supernatants were concentrated by ultrafiltration using Amicon Ultra-15 centrifugal filter units with 50 kDa cut-off filters (EMD Millipore, Burlington, MA, USA) and centrifuging at 3500 rpm (2867× *g*) for 30 min at 4 °C.
(6) Next, the concentrated supernatants (2–4 mL) were used for EV isolation either by the ultracentrifugation method or the immunoprecipitation method as described below.

Ultracentrifugation method: The concentrated supernatants were subjected to ultracentrifugation at 170,000× *g* for 90 min at 4 °C. The supernatant was discarded and the pellet of EVs was resuspended in 100 µL filtered 1X PBS.

Precipitation method: EVs in the concentrated supernatant were precipitated by adding an exosome isolation reagent (Invitrogen, cat# 4478359) at half the volume of the concentrate, as per the manufacturer’s instructions. It was mixed well by vortexing and stored in the refrigerator at 4 °C overnight. The next day, the mixture was centrifuged at 10,000× *g* for 30 min at 4 °C. The supernatant was discarded, and the EV pellet was collected by resuspending in 100 µL filtered 1X PBS. 

### 4.3. Characterization of EVs

#### 4.3.1. Nanoparticle Tracking Analysis (NTA)

The NanoSight NS300 instrument (Malvern Instruments, Malvern, UK) was used to detect EVs. The NanoSight polystyrene latex calibration beads, 100 nm and 200 nm were applied to check the instrument’s performance. In this study, diluted suspensions containing EVs were loaded into the sample chamber, and the camera level was maintained at 10 for light scatter mode between samples. Three videos of typically 30 s duration were taken, with a frame rate of 30 frames per second. Data were analyzed by NTA 3.0 software (Malvern Instruments) which was optimized to first identify and then track each particle on a frame-by-frame basis.

#### 4.3.2. Western Blot

Proteins from EcN-EVs and LA-EVs were extracted using lysis buffer (Thermo Scientific, FL, USA) containing protease inhibitors. Protein concentrations were measured by Bradford assay kit (Bio-Rad Laboratories, Hercules, CA, USA) with a linear range of the assay for BSA between 0.2 and 0.9 mg/mL. Using a spectrofluorometer (BioTek Instruments, Charlotte, VT, USA), absorbance was measured at 595 nm. For Western blot analysis, the proteins underwent electrophoresis and transferred onto PVDF membranes. The membranes were blocked by incubating with 5% dry milk for 1 h before being incubated overnight at 4 °C with primary antibodies. The primary antibodies used included anti-CD63 (Abcam, cat# 217345), anti-TSG-101 (ProteinTech, cat# 14497-1-AP), anti-OMP-A (Antibody Research Corp, cat# 111120), anti-LTA (Abcam, cat# ab267414), and GAPDH (Thermo Fisher, Cat # MA5-15738). After thorough washing, membranes were treated with horseradish peroxidase-conjugated IgG (Jackson ImmunoResearch Labs, West Grove, PA, USA) for 2 h at room temperature. Blots were visualized using enhanced chemiluminescence developing solutions.

### 4.4. Statistical Analysis

Image Lab 5.2.1 (BioRad Laboratories, Hercules, CA, USA), Prism 8.0 (GraphPad Software, San Diego, CA, USA), and Excel version 16.85 (Microsoft, Redmond, WA, USA) software were used for data acquisition, analysis, and presentation. One-way and multifactorial analysis of variance (ANOVA) tests were used for the data analysis in Prism to compare various data sets. Error bars indicated ± standard error of mean (SEM). Probability values of *p* < 0.05 were considered statistically significant.

## 5. Conclusions

Our data showed that ultracentrifugation (combined with ultrafiltration to concentrate the supernatant) is highly suitable for isolating EVs from Gram-positive and Gram-negative probiotic bacteria. The detailed protocol described here is expected to support the large-scale isolation of bacterial EVs for obtaining consistent results in pre-clinical experimental models.

## Data Availability

The original contributions presented in this study are included in the article. Further inquiries can be directed to the corresponding author.
